# A Time for Celebration: 40th Anniversary of GSC and 15th Anniversary of BIG, CAS

**DOI:** 10.1016/j.gpb.2019.02.001

**Published:** 2019-02-08

**Authors:** Jun Yu

**Affiliations:** 1 CAS Key Laboratory of Genome Sciences and Information, Beijing Institute of Genomics, Chinese Academy of Sciences, Beijing 100101, China; 2 University of Chinese Academy of Sciences, Beijing 100049, China

This special collection of *Genomics, Proteomics & Bioinformatics* (GPB) is dedicated to anniversary celebrations of two organizations, of which GPB is an official journal under their auspices. One is the Genetics Society of China (GSC), which was established 40 years ago, and the other is the Beijing Institute of Genomics (BIG), Chinese Academy of Sciences (CAS), which was established in 2003 when the Human Genome Project — a landmark internationally-coordinated project of life sciences — announced the completion of the human genome sequence. And just so happened 15 years ago, our journal, GPB, as well as its editorial and personnel management responsibilities, was also transferred to BIG from another CAS institute. With a new and rather inclusive name, GPB had been subsequently revitalized to cover the three fields specified and has, after a long-term struggle to survive and to flourish, now become one of the major journals internationally in the fields of not only genomics, proteomics, and bioinformatics, but also genetics. Another expansion into broader territories of omics is now inevitable.

For such anniversary celebrations, we publish in this issue three articles on rice as a snapshot to showcase the research progresses achieved by geneticists in China in the past decades. These include one article that looks into historic and landmark achievements of rice genomics [1] and another that provides current status and future prospective of plant genomics and newly developed molecular tools in crop breeding and improvement systems [2]. As a highlight, the seminal work on a groundbreaking genetic discovery leading to elite hybrid rice was recognized by the 2018 China’s Future Science Prize [3]. In addition, we also publish a recent work from alumni of BIG on another very important grass crop, maize, for the identification of genes involved in kernel development [4].

Furthermore, to add a visual attraction, we designed a cover accompanying the featured articles, with a composite from two original paintings, one illustrating rice harvesting and the other demonstrating fabric weaving from the Yinzhen (Yongzheng Emperor of the Qing Dynasty, 1678–1735) Farming and Weaving Illustrations.

We would also like to take this opportunity to invite suggestions and discussions on new territories and landscapes for GPB to thrive further into the future. We envisage that GPB will become a monthly journal as we have been receiving so many more submissions than what we could possibly publish as a bimonthly journal. Therefore, it is time for all of us, the editorial staff and faithful readers and contributors of GPB, to think of ways and actions for the future strategic development of GPB. Some of the ideas include inviting more international members for our editorial board, recruiting new expertise from the leading-edge research activities of GPB-covered fields, and further improving professional recognitions by scientists of the relevant research fields. So, let us put our efforts together to make GPB a successful journal that best serves the research community of genomics, proteomics, bioinformatics, and others to come.
